# Effect of Neoadjuvant Chemoradiotherapy with Capecitabine versus Fluorouracil for Locally Advanced Rectal Cancer: A Meta-Analysis

**DOI:** 10.1155/2016/1798285

**Published:** 2016-11-07

**Authors:** Guo-Chen Liu, Jun-Ping Yan, Qing He, Xin An, Zhi-Zhong Pan, Pei-Rong Ding

**Affiliations:** ^1^Department of Gynecologic Oncology, Sun Yat-sen University Cancer Center, State Key Laboratory of Oncology in South China, Collaborative Innovation Center for Cancer Medicine, Guangdong 510060, China; ^2^Department of Laboratory Medicine, Guangdong No. 2 Provincial People's Hospital, Guangdong, China; ^3^Department of General Surgery, People's Hospital of Yuxi City, Yunnan, China; ^4^Department of Medical Oncology, Sun Yat-sen University Cancer Center, State Key Laboratory of Oncology in South China, Guangdong, China; ^5^Department of Colorectal Surgery, Sun Yat-sen University Cancer Center, State Key Laboratory of Oncology in South China, Guangdong 510060, China

## Abstract

A meta-analysis was carried out to compare the efficacy and safety of capecitabine plus radiation with 5-fluorouracil (5-FU) plus radiotherapy (RT) as neoadjuvant treatment in locally advanced rectal cancer (LARC). We searched the Cochrane database, Ovid, Medline, Embase, ISI databases, and Chinese Biomedical Literature Database between January 1998 and October 2014. Trials of capecitabine compared with 5-FU plus RT as neoadjuvant treatment for LARC were considered for inclusion. RevMan software was used to analyze these data. Nine trials were included in this meta-analysis, which covered a total of 3141 patients. The meta-analysis showed that capecitabine group had statistically significant better pCR rates (OR, 1.34; 95% CI, 1.10–1.64; *P* = 0.003), T downstaging rates (OR, 1.58; 95% CI, 1.22–2.06; *P* = 0.0007), N downstaging rates (OR, 2.06; 95% CI, 1.34–3.16; *P* = 0.001), less distant metastasis (OR, 0.63; 95% CI, 0.44–0.88; *P* = 0.007), and lowered leucocytes (OR, 0.25; 95% CI, 0.11–0.54; *P* = 0.0005), but with higher incidence of hand-foot syndrome (HFS) (OR, 4.43; 95% CI, 1.59–12.33; *P* = 0.004). Capecitabine was more efficient than 5-FU in terms of tumor response in neoadjuvant treatment for patients with LARC and favourably low toxicity with the exception of HFS.

## 1. Introduction

Neoadjuvant chemoradiotherapy with fluoropyrimidine is the standard treatment for locally advanced rectal cancer (LARC), as supported by results of the randomized phase III study conducted by the German Rectal Cancer Study Group [1]. 5-FU combined with leucovorin (LV) is the most commonly administered concurrent chemotherapy. Modifications of perioperative fluorouracil treatment have been investigated in many Phase II and Phase III trials in an attempt to improve overall survival and disease-free survival rate. Compared to bolus 5-FU and pelvic radiotherapy (RT), patients who received concurrent RT with protracted 5-FU infusion had an improved period of time to relapse and postoperative survival in both preoperative [2–4] and postoperative chemoradiotherapy [5, 6]. Therefore, protracted infusion 5-FU is accepted as the standard regimen for concurrent chemoradiotherapy for rectal cancer at many institutions. However, protracted 5-FU infusion requires an indwelling venous catheter, which is associated with increased complications (infection, bleeding, thrombosis, and pneumothorax) [7] and patients' discomfort.

Capecitabine, an orally administered fluoropyrimidine, was shown to have antitumor activity in metastatic colorectal cancer, and it is also more convenient to use [8]. In several randomized trials, capecitabine had been shown to be at least equivalent in efficacy to 5-FU in metastatic colorectal cancer (mCRC) [9, 10]. Capecitabine is preferentially converted to 5-FU in tumor tissue through a three-step enzymatic pathway with the participation of thymidine phosphorylase. X-ray irradiation was found to induce the synthesis of thymidine phosphorylase, providing a rationale that the usage of capecitabine combined with RT might be associated with an improved therapeutic index in patients with cancer [11].

Up to now, only two randomized trials [12, 13] and several retrospective studies [14–20] had compared capecitabine versus 5-FU/LV in neoadjuvant chemoradiotherapy regimens for patients with LARC; however, in terms of pathologic complete response (pCR) rate and toxicities, the results of these studies were not consistent. Therefore, we carried out a meta-analysis to determine the difference in efficacies and toxicities of these two regimens when used as treatment combination with RT in patients with LARC.

## 2. Methods

### 2.1. Literature Search

The Cochrane database, Ovid, Medline, Embase, ISI databases, and Chinese Biomedical Literature Database were searched from January 1998 up to October 2008. The following Mesh search headings were used: “rectal neoplasms”, “neo-adjuvant therapy”, “chemoradiotherapy”, “capecitabine”, and “fluorouracil”. All relevant Oncology Meetings Proceedings (ASCO, AACR, and ASCO GI) and bibliographies of references and reviews were also identified. Furthermore, we searched the reference lists of retrieved relevant articles in order to broaden the scope, and all abstracts, studies, and citations scanned were reviewed too. There were no language restrictions.

### 2.2. Inclusion and Exclusion Criteria

Trials meetings which have the following two criteria were included: (1) compared capecitabine to 5-FU (with or without LV) as neoadjuvant treatment for LARC and (2) illustrated at least one of the outcome measures: tumor response rate, sphincter preservation rate, or adverse effects of treatment/toxicity. When two studies were reported by the same institution, either the one with better quality or the most recent publication was included in this analysis.

Studies were excluded from the analysis if the outcomes of interest were not reported or it was impossible to calculate these from the published results.

### 2.3. Data Extraction

Two researchers assessed all abstracts identified by the above search strategies for subject relevance, respectively. The full publications of all possibly relevant abstracts were obtained and formally assessed for inclusion. Two reviewers independently extracted the following information from each included study: first author, year of publication, characteristics of study population, study design, pathological response to chemoradiation, sphincter preservation, and adverse effects. Adverse effects of treatment/toxicities (grade 3 or 4) data were collected according to National Cancer Institute Common Toxicity Criteria (NCI-CTC v4.0).

### 2.4. Statistical Methods

Statistical analyses were carried out using Review Manager Version 5.0 software (Nordic Cochrane Centre, Copenhagen, Denmark). We calculated the odds ratio (OR) for dichotomous data with 95% confidence intervals (CIs) for all analyses. Two techniques were used to calculate the pooled OR estimates: the Mantel-Haenszel method [21], which assumes a fixed-effects model, and the DerSimonian-Laird method [22], which assumes a random-effects model. If there was no heterogeneity, the fixed- and the random-effects models provided similar results, and we chose fixed-effects model. When heterogeneity was found, the random-effects model was considered to be more appropriate, although both models might be biased. An estimation of disease-free survival based on Kaplan-Meier plots was performed by DigitizeIt software 2.06 (Bormann, bormisoft@digitizeit.de).

Statistical heterogeneity between studies was assessed by the chi-squared test with significance set at a *P* value of 0.10 and the quantity of heterogeneity measured using the *I* ^2^ statistic. Negative values of *I* ^2^ were put equal to zero, so that *I* ^2^ lay between 0% and 100%. A value of 0% indicates no observed heterogeneity, and larger values show increasing heterogeneity. Publication bias was assessed by use of a funnel plot [23].

## 3. Results

### 3.1. Description of Studies

**Figure 1:**
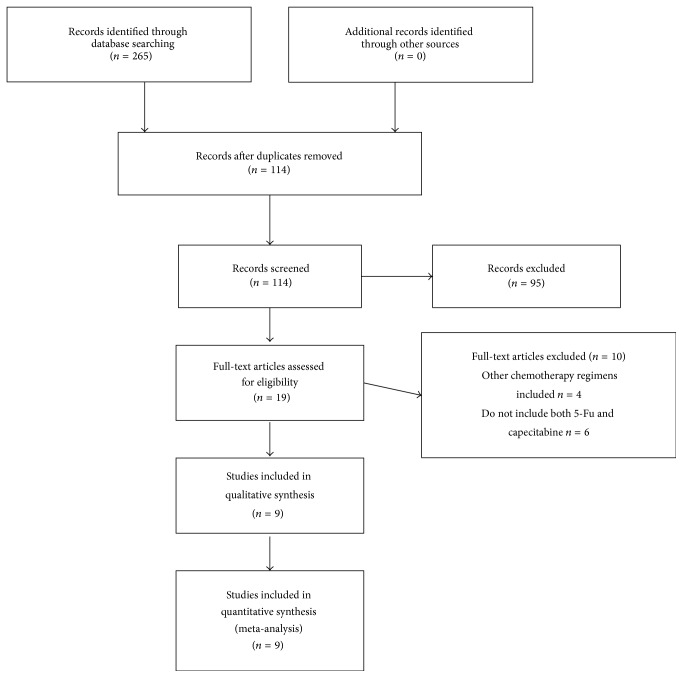
The Quality of Reporting of Meta-Analyses (QUOROM) statement flow diagram.

Figure 1 showed the process used to select potentially relevant studies for inclusion; 114 records were identified by the primary computerized literature search. However, after screening the titles and abstracts, we excluded 95 studies which were either case series, review articles (*n* = 40), or irrelevant to the current study (*n* = 55). The full texts of the retrieved 19 manuscripts were clearly read, and the reference lists were checked. After excluding 4 articles including other regimens and 6 studies which did not include both 5-FU and capecitabine simultaneously, finally, we included the remaining 9 trials in the meta-analysis [12–20], having covered a total of 3141 patients. Among these patients, 1541 pts were found to be treated with capecitabine and 1600 pts with 5-FU as neoadjuvant chemotherapy regimen. It should be noted that one trial [13] alone out of the 9 trials contributed almost one-half of the patients included in the present meta-analysis. The characteristics of the nine studies included in this paper were shown in Table 1, two of them were prospective studies [12, 13] with a combined total of 2000 subjects, and 7 were retrospective studies which covered a total of 1141 patients [14–20].

It should be noted that the NSABP-R-04 protocol [13] was a two-step study, (1) from September 2004 to October 2005, 293 patients were randomly assigned in the two-arm portion of the study (capecitabine versus 5-FU) and (2) from October 2005 to August 2010, additional 1315 patients were randomly assigned into four-arm study: 330 patients to RT plus 5-FU, 329 patients to RT plus 5-FU and oxaliplatin, 326 patients to RT plus capecitabine, and 330 patients to RT plus capecitabine and oxaliplatin. The author did not mention the pCR rates of patients received capecitabine/5-FU monotherapy plus RT. However, in this protocol, there was no significant detrimental effect on pCR rates when neoadjuvant chemotherapy was combined with oxaliplatin or not (*P* = 0.42). Therefore, all 1608 patients were included in the analysis for pCR rates.

### 3.2. Tumor Response

**Figure 2:**
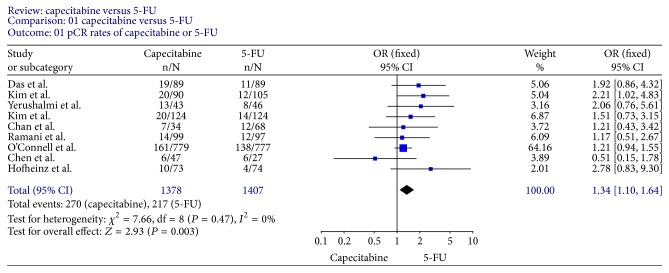
Forest plot of odds ratio (OR) for pCR rate of all trials.

**Figure 3:**
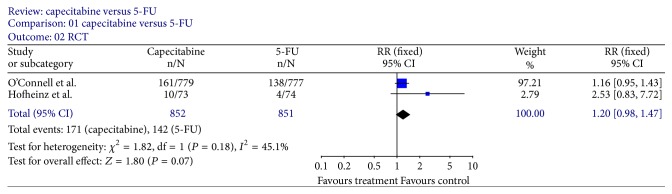
Forest plot of relative risk (RR) for pCR rate of RCTs only.

**Figure 4:**
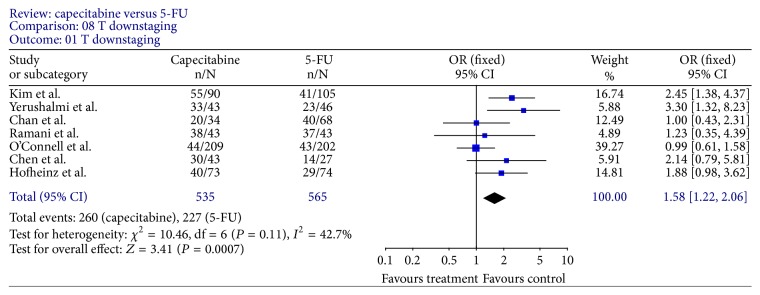
Forest plot of odds ratio (OR) for T downstaging rate.

**Figure 5:**
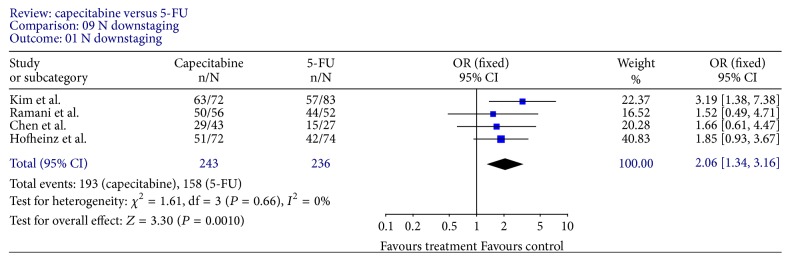
Forest plot of odds ratio (OR) for N downstaging rate.

Tumor response rates were reported in all trials. All 9 trials reported pCR rates (Figure 2); the fixed-effect pooled estimate including 2785 patients evaluated for OR showed a statistically significant increased OR for capecitabine (OR, 1.34; 95% CI, 1.10–1.64; *P* = 0.003). The inclusion of the two RCTs only to this analysis led to an RR of 1.20 (95% CI, 0.98–1.47; *P* = 0.07) (Figure 3). Seven trials [12–15, 18–20] reported T downstaging (the reduction of the T stage by at least one level after neoadjuvant chemoradiation); random-effect pooled estimate demonstrated an increased OR for capecitabine (OR, 1.58; 95% CI, 1.22–2.06; *P* = 0.0007) (Figure 4). However, there were heterogeneities between these trials (*I* = 53%, *P* = 0.05). Four trials [12, 15, 18, 19] reported N downstaging (the reduction of the N stage by at least one level after neoadjuvant chemoradiation); random-effect pooled estimate demonstrated an increased OR for capecitabine as well (OR, 2.06; 95% CI, 1.34–3.16; *P* = 0.001) (Figure 5).

### 3.3. Type of Surgical Resection

**Figure 6:**
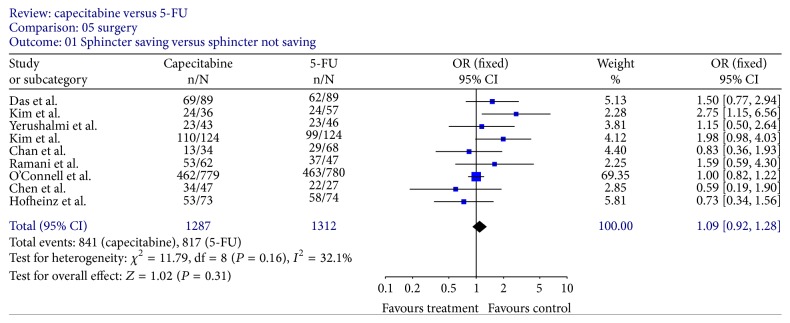
Forest plot of odds ratio (OR) for sphincter-sparing resection rate.

Data on the rate of sphincter-sparing resection were available for all the 9 trials; no statistically significant difference was demonstrated between the two regimens (OR, 1.09; 95% CI, 0.92–1.28; *P* = 0.31) (Figure 6).

### 3.4. Recurrence

**Figure 7:**
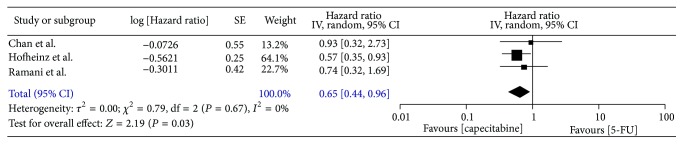
Forest plot of hazard ratio (HR) for 3-year disease-free survival.

**Figure 8:**
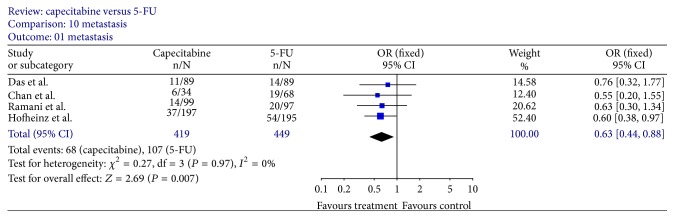
Forest plot of odds ratio (OR) for metastasis rate.

**Figure 9:**
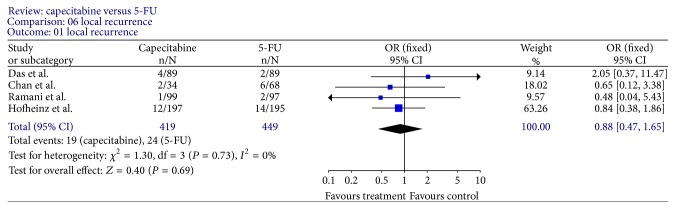
Forest plot of odds ratio (OR) for local recurrence rate.

Four trials [12, 14, 16, 19] provided data for disease-free survival; however, Kaplan-Meier plots of disease-free survival were only available in 3 trials [12, 14, 19], including 690 patients (capecitabine, *n* = 330; 5-FU, *n* = 360). The meta-analysis of the pooled data demonstrated that the 3-year disease-free survival was in favour of capecitabine-based regimens (HR, 0.65; 95% CI, 0.44–0.96, *P* = 0.03; Figure 7). 16.2% (68/419) patients in capecitabine group and 23.8% (107/449) in 5-FU group had distant recurrence, and there was significant difference (OR, 0.63; 95% CI, 0.44–0.88; *P* = 0.007, Figure 8). However, there was no significant difference in the local recurrence rate between the two groups (OR, 0.88; 95% CI, 0.47–1.65; *P* = 0.69, Figure 9).

### 3.5. Toxicity

With regard to toxicity, we chose the most frequently reported side-effects (at least mentioned in four trials) to obtain reliable comparisons between capecitabine and 5-FU regimen. Thus, toxicity was not evaluated completely and the reported results must be interpreted with caution. The adverse events included lowered hemoglobin, lowered leucocytes, lowered platelets, nausea, vomiting, diarrhea, mucositis, hand-foot syndrome, and radiation dermatitis. The most frequent toxicity was diarrhea in patients receiving either capecitabine (87/1204) or 5-FU (95/1252) combined with RT (Table 2).

**Figure 10:**
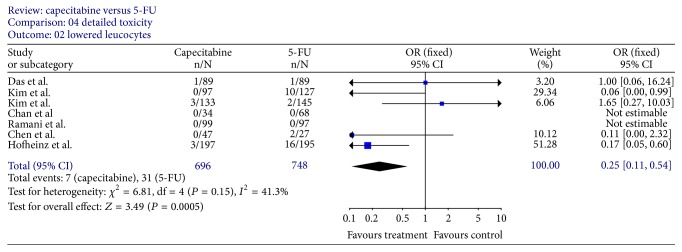
Forest plot of odds ratio (OR) for lowered leucocytes rate.

**Figure 11:**
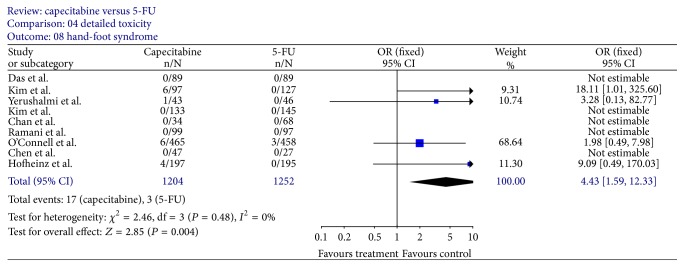
Forest plot of odds ratio (OR) for hand-foot syndrome rate.

Grade 3 or worse lowered leucocytes (reported by 7 trials [12, 14–19]) were significantly less prominent in patients receiving capecitabine and RT (OR, 0.25; 95% CI, 0.11–0.54; *P* = 0.0005) (Figure 10). Moreover, after excluding the only trial [17] in which capecitabine group had more patients with lowered leucocytes, the difference was more obvious (OR 0.08; 95% CI, 0.02 to 0.30; *P* = 0.0002) with less heterogeneity (*P* = 0.48). However, the frequency of hand-foot syndrome (reported by all trials) appeared higher in the capecitabine and RT group (OR, 4.43; 95% CI, 1.59–12.33; *P* = 0.004) (Figure 11). A sensitivity analysis showed that several adverse effects were not statistically different between capecitabine and 5-FU, such as lowered hemoglobin [12, 14–19], lowered platelets [12, 16–20], nausea [12–15, 18, 19], vomiting [12, 13, 15, 18, 19], mucositis [12, 14, 16, 19], and radiation dermatitis [12–16, 18, 20], whereas, with respect to severe diarrhea (reported by 7 trials [12–20]), the findings of major heterogeneity between these trials (*I* = 64.5% and *P* = 0.004) restricted a valid interpretation of the pooled estimates.

### 3.6. Evaluation of Publication Bias

**Figure 12:**
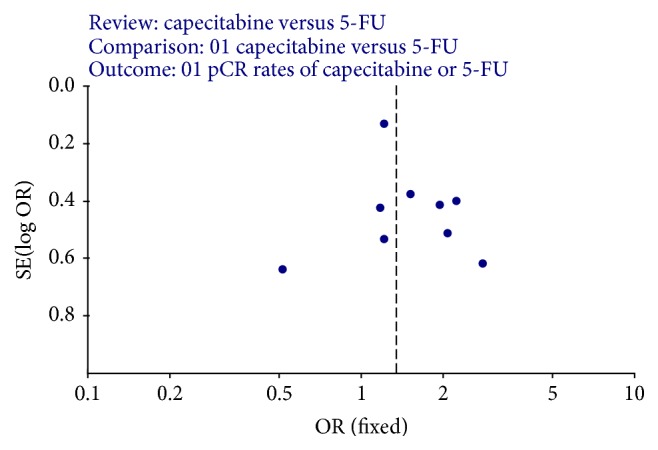
Funnel plot of studies included in this meta-analysis.

A funnel plot of the studies used in the meta-analysis reporting on capecitabine versus 5-FU as chemotherapy plus RT used as neoadjuvant treatment for patients with LARC was shown in Figure 12. There was no evidence of publication bias (all studies were equally distributed round the vertical axis).

## 4. Discussion

This meta-analysis compared the efficacy and safety of capecitabine plus radiation with 5-FU plus RT as neoadjuvant treatment in LARC. In the light of its favourable efficacy and low toxicity, the analysis showed that capecitabine was superior to 5-FU in neoadjuvant treatment for patients with LARC. Capecitabine plus RT might be regarded as standard neoadjuvant treatment in patients with LARC considering its advantage.

The previous work by Meta-Analysis Group in Cancer proved continuous intravenous infusion (CIV) 5-FU is superior to bolus 5-FU in terms of tumor response based on other studies of advanced colorectal cancer [24]. Capecitabine mimics CIV 5-FU in its pharmacologic action in vivo; it has shown equivalent efficacy to CIV 5-FU plus leucovorin on metastatic colorectal cancer, but without the use of venous catheter and better patient tolerability. Both the two included RCT trials confirmed the equivalence of CIV 5-FU and capecitabine in neoadjuvant chemoradiotherapy regimens for patients with LARC. However, regarding outcome and toxicity evaluation, heterogeneities still remained between different studies.

This meta-analysis brought together all currently available data comparing capecitabine with 5-FU plus RT used as neoadjuvant treatment for patients with LARC.

Meta-analysis of all studies showed a significantly better pCR, T downstaging, N downstaging, when radiation therapy is combined with capecitabine instead of 5-FU for the neoadjuvant treatment of LARC. As far as the adverse events were concerned, there appeared to be higher rate of hand-foot syndrome and lower rate of lowered leucocytes in the capecitabine arm. However, the type of surgical resection and other adverse events (lowered hemoglobin, lowered platelets, nausea, vomiting, diarrhea, mucositis, and radiation dermatitis) were not statistically different between these two regimens. Since the data on diarrhea, radiation dermatitis were different between the pooled trials; they should be interpreted with caution.

Only four trials mentioned long-term survivorship; interestingly, in all studies, capecitabine showed reduction in distant metastases and noninferiority in both DFS and OS, suggesting greater systemic efficacy than with 5-FU, in accordance with the X-ACT study comparing capecitabine with bolus fluorouracil plus folinic acid for adjuvant treatment of stage III colon cancer. This could be partly explained by the higher pCR rates of capecitabine group. As proved by other trials [25], patients with a pCR after chemoradiation have a significantly better long-term outcome than do those with residual disease, although this could be influenced by adjuvant treatment; additional evidence will be required to assess the long-term prognosis of capecitabine and 5-FU in the preoperative combined-modality therapy of LARC.

Hand-foot syndrome is predominant in capecitabine treatment group but could be easily managed with vitamin B6 administration and supportive care without interrupting the RT schedule. Interestingly, in the trial of Hofheinz et al., those patients who developed HFS enjoyed better DFS and overall survival than those who did not develop in both capecitabine group and the overall study population [12].

Our study had several limitations, and the efficiency of this meta-analysis might have been affected by several factors. First of all, because of the possibility that not all relevant studies were identified by computerized searching, it was necessary to combine the electronic material with manual research and personal contact with authors. Unfortunately, efforts on this were not very productive. Secondly, only two RCTs were included in this meta-analysis, and the number of patients in NSABP-R-04 accounts for almost one-half; the small sample sizes of the retrospective trials included might affect the conclusion. Thirdly, the usage of oxaliplatin may also influence the pCR rate in the NSABP-R-04 trial. Finally, varying doses and application of capecitabine and 5-FU might influence the results.

In conclusion, as a convenient oral fluoropyrimidine, capecitabine seemed to be at least as good as continuous 5-FU and was an acceptable alternative in the neoadjuvant chemoradiation treatment of patients with LARC. It was associated with improvement tumor response rate and favourably low toxicity with the exception of HFS. Based on these results, we would recommend the use of capecitabine combined with pelvic radiation as a neoadjuvant treatment for LARC.

## Figures and Tables

**Table 1:** Characteristics of the nine studies included in this paper.

Trials	Year	Type of study	Number of pts	Number of pts		Dose of medicine				Dose of RT	Time interval^&^	Results					
				Cape	5-Fu	Cape	DI (mg/m^2^/w)	5-Fu	DI (mg/m^2^/w)			pCR	SSS	SD	OS	DFS	LR
Hofheinz et al. [12]	2012	RCT	392	197	195	2500 mg/m^2^/day days_1–14_ repeated day_22_ (2 cycles before and after RT) 1650 mg/m^2^/day (during RT)	10113	500 mg/m^2^/day days_1–5_ repeated day_22_ (2 cycles before and after RT) 225 mg/m^2^/day (during RT)	966	50.4 Gy/25 Fr	4–6 weeks	*✓*	*✓*	*✓*	*✓*	*✓*	*✓*

O'Connell et al. [13]	2014	RCT	1,608	802	806	1650 mg/m^2^/day 5 days/weeks_1–5_	8250	225 mg/m^2^/day 5 days/weeks_1–5_	1125	T3N0: 50.4 Gy/25 Fr; T4/N+: 55.8 Gy/25 Fr	6–8 weeks	*✓*	*✓*	*✓*	*✗*	*✗*	*✗*

Chen et al. [15]	2012	Retro	74	47	27	1700 mg/m^2^/day 5 days/weeks_1–5_	8500	350 mg/m^2^/day 5 days/weeks_1,5_	700	45–50.4 Gy/25 Fr,	6 weeks	*✓*	*✓*	*✓*	*✗*	*✗*	*✗*

Chan et al. [14]	2010	Retro	102	34	68	1650 mg/m^2^/day 5 days/weeks_1–5_	8250	20 mg/kg/day days_1–4,22–25_	N/A	50 Gy/25 Fr	8 weeks	*✓*	*✓*	*✓*	*✓*	*✓*	*✓*

Yerushalmi et al. [20]	2006	Retro	89	43	46	1650 mg/m^2^/day 5 days/weeks_1–5_	8250	180 mg/m^2^/day 5 days/weeks_1–5_	900	50 Gy/25 Fr	7–7.5 weeks (median)	*✓*	*✓*	*✓*	*✗*	*✗*	*✗*

Das et al. [16]	2006	Retro	178	89	89	1650 mg/m^2^/day 5 days/weeks_1–5_ 58 pts; 1650 mg/m^2^/day 7 days/weeks_1–5_ 31 pts	9399	300 mg/m^2^/day 5 days/weeks_1–5_	1500	52.5 Gy/25 Fr	N/A	*✓*	*✓*	*✓*	*✓*	*✓*	*✓*

Kim et al. [17]	2007	Retro	278	133	145	1650 mg/m^2^/day 7 days/weeks_1–5_	11550	400 mg/m^2^/day 3 days/weeks_1,5_	480	50 Gy/25 Fr	6 weeks	*✓*	*✓*	*✓*	*✗*	*✗*	*✗*

Ramani et al. [19]	2010	Retro	196	99	97	1650 mg/m^2^/day 5 days/weeks_1–5_	8250	1000 mg/m^2^/day days_1–4,22–26_	1600	50.4–54 Gy/25 Fr	6–8 weeks	*✓*	*✓*	*✓*	*✓*	*✓*	*✓*

Kim et al. [18]	2006	Retro	224	97	127	1650 mg/m^2^/day days_1–14_ repeated day_22_	9240	500 mg/m^2^/day 5 days/weeks_1,5_	1000	50.4 Gy/25 Fr	6 weeks	*✓*	*✓*	*✓*	*✗*	*✗*	*✗*

RCT, randomized clinical trial; retro, retrospective study; number of pts, number of patients; cape, capecitabine; RT, radiotherapy; LR, local recurrence; pCR, pathological complete response; SSS, sphincter saving surgery; OS, overall survival; DFS, disease-free survival; SD, surgical downstaging; DI, dose intensity; RR, risk ratio; CI, confidence interval.

&: time interval between radiotherapy and surgery.

**Table 2:** Toxic (grade 3 or worse^*∗*^) effects in trials comparing capecitabine with 5-Fu.

Toxicity	Number of trials	Number of cases		OR (95% CI)	Test of homogeneity		*P* value
		Cape	5-Fu		*I* ^2^ (%)	*P* value	
Lowered hemoglobin	7	6/696	5/748	1.13 (0.36, 3.54)^#^	24.6	0.27	0.83
Lowered leucocytes	7	7/696	31/748	0.24 (0.11, 0.54)^#^	28.9	0.23	0.0005
Lowered platelets	6	0/658	4/699	0.30 (0.05, 1.88)^#^	0	0.99	0.2
Nausea	6	10/906	4/905	2.30 (0.79, 6.70)^#^	0	0.51	0.13
Vomiting	6	7/1038	2/1049	3.04 (0.72, 12.75)^#^	0	0.66	0.13
Diarrhea	9	87/1204	95/1252	0.92 (0.67, 1.24)^§^	66.7	0.002	0.57
Mucositis	4	1/419	4/449	0.33 (0.05, 2.10)^#^	0	0.64	0.24
Hand-foot syndrome	9	17/1204	3/1252	4.43 (1.59, 12.33)^#^	0	0.48	0.004
Radiation dermatitis	7	26/972	32/1010	0.85 (0.51, 1.44)^§^	49.3	0.08	0.55

RR, risk ratio; cape, capecitabine; CI, confidence interval.

^#^Fixed-effect model.

^§^Random-effects model.

^*∗*^National Cancer Institute Common Terminology Criteria for Adverse Events, version 4.0.

## References

[1] Sauer R., Becker H., Hohenberger W. (2004). Preoperative versus postoperative chemoradiotherapy for rectal cancer. *The New England Journal of Medicine*.

[2] Rich T. A., Skibber J. M., Ajani J. A. (1995). Preoperative infusional chemoradiation therapy for Stage T3 rectal cancer. *International Journal of Radiation Oncology, Biology, Physics*.

[3] Janjan N. A., Crane C. N., Feig B. W. (2000). Prospective trial of preoperative concomitant boost radiotherapy with continuous infusion 5-fluorouracil for locally advanced rectal cancer. *International Journal of Radiation Oncology Biology Physics*.

[4] Crane C. H., Skibber J. M., Birnbaum E. H. (2003). The addition of continuous infusion 5-FU to preoperative radiation therapy increases tumor response, leading to increased sphincter preservation in locally advanced rectal cancer. *International Journal of Radiation Oncology Biology Physics*.

[5] O'Connell M. J., Martenson J. A., Wieand H. S. (1994). Improving adjuvant therapy for rectal cancer by combining protracted-infusion fluorouracil with radiation therapy after curative surgery. *The New England Journal of Medicine*.

[6] Kalofonos H. P., Bamias A., Koutras A. (2008). A randomised phase III trial of adjuvant radio-chemotherapy comparing Irinotecan, 5FU and Leucovorin to 5FU and Leucovorin in patients with rectal cancer: a Hellenic Cooperative Oncology Group Study. *European Journal of Cancer*.

[7] Di Carlo I., Pulvirenti E., Mannino M., Toro A. (2010). Increased use of percutaneous technique for totally implantable venous access devices. Is it real progress? A 27-year comprehensive review on early complications. *Annals of Surgical Oncology*.

[8] Mayer R. J. (2007). Should capecitabine replace infusional fluorouracil and leucovorin when combined with oxaliplatin in metastatic colorectal cancer?. *Journal of Clinical Oncology*.

[9] Van Cutsem E., Findlay M., Osterwalder B. (2000). Capecitabine, an oral fluoropyrimidine carbamate with substantial activity in advanced colorectal cancer: results of a randomized phase II study. *Journal of Clinical Oncology*.

[10] Hoff P. M., Ansari R., Batist G. (2001). Comparison of oral capecitabine versus intravenous fluorouracil plus leucovorin as first-line treatment in 605 patients with metastatic colorectal cancer: results of a randomized phase III study. *Journal of Clinical Oncology*.

[11] Dunst J., Reese T., Sutter T. (2002). Phase I trial evaluating the concurrent combination of radiotherapy and capecitabine in rectal cancer. *Journal of Clinical Oncology*.

[12] Hofheinz R.-D., Wenz F., Post S. (2012). Chemoradiotherapy with capecitabine versus fluorouracil for locally advanced rectal cancer: a randomised, multicentre, non-inferiority, phase 3 trial. *The Lancet Oncology*.

[13] O'Connell M. J., Colangelo L. H., Beart R. W. (2014). Capecitabine and oxaliplatin in the preoperative multimodality treatment of rectal cancer: surgical end points from national surgical adjuvant breast and bowel project trial R-04. *Journal of Clinical Oncology*.

[14] Chan A. K., Wong A. O., Jenken D. A. (2010). Preoperative capecitabine and pelvic radiation in locally advanced rectal cancer—is it equivalent to 5-FU infusion plus leucovorin and radiotherapy?. *International Journal of Radiation Oncology, Biology, Physics*.

[15] Chen C.-F., Huang M.-Y., Huang C.-J. (2012). A observational study of the efficacy and safety of capecitabine versus bolus infusional 5-fluorouracil in pre-operative chemoradiotherapy for locally advanced rectal cancer. *International Journal of Colorectal Disease*.

[16] Das P., Lin E. H., Bhatia S. (2006). Preoperative chemoradiotherapy with capecitabine versus protracted infusion 5-fluorouracil for rectal cancer: a matched-pair analysis. *International Journal of Radiation Oncology Biology Physics*.

[17] Kim D. Y., Jung K. H., Kim T. H. (2007). Comparison of 5-fluorouracil/leucovorin and capecitabine in preoperative chemoradiotherapy for locally advanced rectal cancer. *International Journal of Radiation Oncology Biology Physics*.

[18] Kim J.-S., Kim J.-S., Cho M.-J., Yoon W.-H., Song K.-S. (2006). Comparison of the efficacy of oral capecitabine versus bolus 5-FU in preoperative radiotherapy of locally advanced rectal cancer. *Journal of Korean Medical Science*.

[19] Ramani V. S., Sun Myint A., Montazeri A., Wong H. (2010). Preoperative chemoradiotherapy for rectal cancer: a comparison between intravenous 5-fluorouracil and oral capecitabine. *Colorectal Disease*.

[20] Yerushalmi R., Idelevich E., Dror Y. (2006). Preoperative chemoradiation in rectal cancer: retrospective comparison between capecitabine and continuous infusion of 5-fluorouracil. *Journal of Surgical Oncology*.

[21] Demets D. L. (1987). Methods for combining randomized clinical trials: strengths and limitations. *Statistics in Medicine*.

[22] DerSimonian R., Laird N. (1986). Meta-analysis in clinical trials. *Controlled Clinical Trials*.

[23] Egger M., Smith G. D., Schneider M., Minder C. (1997). Bias in meta-analysis detected by a simple, graphical test. *The British Medical Journal*.

[24] Piedbois P., Rougier P., Buyse M. (1998). Efficacy of intravenous continuous infusion of fluorouracil compared with bolus administration in advanced colorectal cancer. *Journal of Clinical Oncology*.

[25] Maas M., Nelemans P. J., Valentini V. (2010). Long-term outcome in patients with a pathological complete response after chemoradiation for rectal cancer: a pooled analysis of individual patient data. *The Lancet Oncology*.

